# Genetic Deletion of Transglutaminase 2 Does Not Rescue the Phenotypic Deficits Observed in R6/2 and zQ175 Mouse Models of Huntington's Disease

**DOI:** 10.1371/journal.pone.0099520

**Published:** 2014-06-23

**Authors:** Liliana B. Menalled, Andrea E. Kudwa, Steve Oakeshott, Andrew Farrar, Neil Paterson, Igor Filippov, Sam Miller, Mei Kwan, Michael Olsen, Jose Beltran, Justin Torello, Jon Fitzpatrick, Richard Mushlin, Kimberly Cox, Kristi McConnell, Matthew Mazzella, Dansha He, Georgina F. Osborne, Rand Al-Nackkash, Gill P. Bates, Pasi Tuunanen, Kimmo Lehtimaki, Dani Brunner, Afshin Ghavami, Sylvie Ramboz, Larry Park, Douglas Macdonald, Ignacio Munoz-Sanjuan, David Howland

**Affiliations:** 1 PsychoGenics Inc., Tarrytown, New York, United States of America; 2 Department of Medical and Molecular Genetics, King's College London, London, United Kingdom; 3 Charles River Discovery Research Services, Kuopio, Finland; 4 CHDI Management/CHDI Foundation, Princeton, New Jersey, United States of America; University of Florida, United States of America

## Abstract

Huntington's disease (HD) is an autosomal dominant, progressive neurodegenerative disorder caused by expansion of CAG repeats in the huntingtin gene. Tissue transglutaminase 2 (TG2), a multi-functional enzyme, was found to be increased both in HD patients and in mouse models of the disease. Furthermore, beneficial effects have been reported from the genetic ablation of TG2 in R6/2 and R6/1 mouse lines. To further evaluate the validity of this target for the treatment of HD, we examined the effects of TG2 deletion in two genetic mouse models of HD: R6/2 CAG 240 and zQ175 knock in (KI). Contrary to previous reports, under rigorous experimental conditions we found that TG2 ablation had no effect on either motor or cognitive deficits, or on the weight loss. In addition, under optimal husbandry conditions, TG2 ablation did not extend R6/2 lifespan. Moreover, TG2 deletion did not change the huntingtin aggregate load in cortex or striatum and did not decrease the brain atrophy observed in either mouse line. Finally, no amelioration of the dysregulation of striatal and cortical gene markers was detected. We conclude that TG2 is not a valid therapeutic target for the treatment of HD.

## Introduction

Tissue transglutaminase 2 (TG2; TGM2; human Gene ID# 7052) is a multi-functional enzyme primarily known for its calcium-dependent intra- and intermolecular cross-linking activity via isopeptide bond formation between glutamine and lysine residues [1]. TG2 has also been shown to have other enzymatic activities, such as GTPase [2], ATPase [3]and isomerase [4] activities. Genetic deletion of TG2 in mice has suggested a role for TG2 activity in mitochondrial energy function [5]. Additionally, TG2 over-activity has been associated with inflammatory diseases such as celiac disease, infectious diseases, cancer, and neurodegenerative diseases such as Huntington's disease (HD) [6]–[9].

HD is a genetic, autosomal dominant, progressive neurodegenerative disorder caused by the expansion of the CAG trinucleotide repeat found in the huntingtin gene; when the repeat number exceeds 39, individuals will develop HD within an normal lifespan [10]. Clinically, the disorder is characterized by motor and cognitive deficits as well as, psychiatric problems. Histopathologically, the disorder is characterized by striatal and cortical atrophy, formation of intracellular huntingtin aggregates, and gene expression changes [10].

TG2 expression and transglutaminase activity have both been demonstrated to be increased in HD patients [11]–[13] and in HD mouse models [14], [15], which suggests a correlation with HD pathology. Additionally, genetic deletion of TG2 in two mouse models of HD, R6/1 and R6/2, resulted in improved phenotypes including reduced neuronal cell death, improved motor performance, and prolonged survival [16], [17]. These reports led us to initiate a medicinal chemistry program targeting the transglutaminase activity of TG2 as a potential therapeutic strategy for the treatment of HD [18], [19]. This program was recently terminated due to the lack of tractability of our TG2 inhibitors to pharmacologically modulate this enzyme in the brain. However, given the existing data derived from the genetic studies, we wanted to extend the validation of this target for the treatment of HD. We therefore repeated this study design with the R6/2 mouse model [20] as well as the zQ175 KI mouse model [21], [22] to investigate the molecular, behavioral and pathophysiological association between TG2 and HD by crossing these two lines with a TG2 knockout (KO) mouse [23]. These KO mice are viable, fertile and do not present any abnormal phenotypes [23]. The behavioural effect of genetic depletion of TG2 was examined using the PhenoCube system, the procedural water T-maze test and a Go/No-go paradigm. In addition, body weight and survival were examined. We also used molecular and imaging techniques to further characterize the phenotypes of the double mutants.

In contrast to previously published findings [16], [17], we report here that the genetic deletion of TG2 in either the R6/2 transgenic or zQ175 KI mice did not improve motor, cognitive, molecular, histological, or lifespan phenotypes, indicating that TG2 expression is not a determinant of disease progression in these models of HD.

## Materials and Methods

### 1. Animals

Animals were bred in the CHDI colonies at the Jackson laboratories. Experimental mice were generated after two successive matings. The R6/2 CAG 240×TG2 KO line (CHDI-80000024-1) was generated by crossing R6/2 CAG 240 (CHDI-80000004-1, R6/2, C57Bl/6J) ovarian-transplanted females with TG2/− (CHDI-80000013-1, C57Bl/6J; [23]) male mice. Dr Graham kindly provided us with the TG2 (+/−) animals to generate the crosses. The R6/2 TG2+/− males offspring generated from that cross were then bred with TG2+/− female mice. From this second cross, we were able to generate littermates of all genotype combinations, namely: R6/2, R6/2 TG2+/−, R6/2 TG2−/−, WT (wild type), TG2+/− (heterozygous TG2 knockout) and TG2−/− (homozygous TG2 knockout). To generate the zQ175×TG2 KO line (CHDI-80000025-1), zQ175 HET (HET, CHDI-80000015-1, C57Bl/6J) females were bred with TG2−/− male mice (CHDI-80000013-1). zQ175 HET TG2+/− females and males generated from that cross were then crossed which allowed the generation of littermates of all genotype combinations, namely: HOM (zQ175 HOM), HOM TG2+/−, HOM TG2−/−, HET (zQ175 HET), HET TG2+/−, HET TG2−/−, WT, TG2+/− and TG2−/−). At 3 weeks of age, animals were weaned by sex and litter, ear tagged for identification and tail samples were collected for genotyping. At 4 weeks of age, animals were pooled into larger groups by sex and genotype. Genotypes were determined by polymerase chain reaction (PCR). CAG repeat lengths were measured by Laragen (Los Angeles, CA, USA) using standard protocols and Genemapper software as previously described [24]. The average CAG repeat length in the R6/2 mice included in the behavioral cohort from the R6/2×TG2 KO line was 247.83 (S.D. = 10.54). The average CAG repeat length of the zQ175 HOM and HET mice from the zQ175×TG2 KO line included in the behavioral cohort was 187.58 (S.D. = 6.05).

### 2. Ethics statement

This study was carried out in strict accordance with the recommendations in the Guide for the Care and Use of Laboratory Animals, NRC (2010). The protocols were approved by the Institutional Animal Care and Use Committee of PsychoGenics, Inc., an AAALAC International accredited institution (Unit #001213).

### 3. Husbandry

At Jackson laboratories, animals were housed in ventilated caging (Thoren Caging System, Inc.) with shaving bedding in 14:10 light-dark cycle.

R6/2×TG2 KO line: animals arrived at PsychoGenics Inc (Tarrytown, NY) at around 4 weeks of age. Mice were transferred from the shipping crates to opti-RAT cages (cage square inches: 144; Animal Care Systems) in the same housing groups as at the Jackson laboratories (6–8 genetically homogenous animals per cage).

zQ175×TG2 KO line: animals arrived at PsychoGenics at around 24/25 weeks of age. Mice assigned to behavioral experiments were transferred from the shipping crates to opti-RAT cages (Animal Care Systems) in the same housing groups as at the Jackson laboratories (4–6 genetically homogenous animals per cage). Animals assigned to molecular experiment were transferred from shipping crates to opti-MICE cages (cage square inches: 75; Animals Care Systems).In all cases, a scope of bedding from the crate was transfer into the opti cage upon arrival. To provide a moderate level of environmental enrichment, opti-Rat contained Enviro-dri bedding, two tunnels and 2 nylon bones while opti-Mice cages contained also Enviro-dri bedding, a tunnel and a nylon bone. Animals had free access to food (Purina 5001) and water unless otherwise specified. Animals were housed under controlled temperature (20°–24°C), humidity (30–70%) and lighting conditions (12:12 light-dark cycle). Animals from the R6/2×TG2 KO line also received supplemental wet feed from 16 weeks of age onwards.

### 4. Body weight

Mice from the R6/2×TG2 KO line and from the zQ175×TG2 KO line were weighed weekly from 4 weeks of age until death and from 24 to 52 weeks of age, respectively.

### 5. Survival

Mice were monitored upon arrival at PsychoGenics routinely, with a minimum of 2 health checks per day. Data were collected for survival analysis which included only animals either found dead or euthanized due to clear morbidity (failure to right themselves after 30 s). Once all the R6/2 mice were deceased (at 26 weeks), all of the remaining WT mice were sacrificed. Animals were euthanized either by decapitation or CO_2_ exposure, following the AVMA Guidelines for Euthanasia for Animals: 2007 Edition.

### 6. Behavioral evaluation

Experimenters conducting behavioral experiments were blind to the genotype. A longitudinal design was used to evaluate the mice from the R6/2×TG2 KO line in the PhenoCube system at around 8, 12 and 16 weeks of age. At 14 weeks of age, mice were evaluated in the procedural water T-maze test. Survival was evaluated in this cohort during the course of the behavioral evaluation. Sample sizes are presented in Table 1.

**Table 1:** Summary of R6/2×TG2 cohorts.

Genotype	WT		TG2+/−		TG2−/−		R6/2		R6/2 TG2+/−		R6/2 TG2−/−	
Sex	M	F	M	F	M	F	M	F	M	F	M	F
**Cohort 1: PhenoCube/T maze, Survival**	16	16	16	15	16	14	14	13	16	16	15	16
**Cohort 2: qPCR analysis - aggregation assay** *	5	6 (3)	6 (4)	6 (4)	7 (4)	5 (4)	6	6	6 (5)	6 (5)	6	6
**Cohort 3: MRI volumetric study**	6	6	6	6	6	6	6	6	6	6	6	6

* Half of the cortex and the striatal tissues collected from each animal were used for real time qPCR analysis.

The other half cortex was used in the aggregation assay. The numbers in brackets indicate the sample size used for in the aggregation assay when it was different from the one used in qPCR studies.

Animals from the zQ175×TG2 KO line were examined in the PhenoCube system at around 27–28 weeks of age. After testing was complete, animals were split in 2 cohorts, one tested on the procedural water T-maze test (28–29 weeks of age) and the other on the Go/No-go operant paradigm from 32 to 34 weeks of age (sample size presented in Table 2).

**Table 2:** Summary of zQ175×TG2 cohorts.

Genotype	WT		TG2+/−		TG2−/−		HET		HET TG2+/−		HET TG2−/−		HOM		HOM TG2+/−		HOM TG2−/−	
Sex	M	F	M	F	M	F	M	F	M	F	M	F	M	F	M	F	M	F
**Cohort 1: PhenoCube**	12	12	0	0	6	12	12	12	0	0	12	18	9	12	0	0	5	7
**Subset A from cohort 1:T maze and MRI volumetric study**	6	6	0	0	6	6	6	6	0	0	6	6	6	6	0	0	5	6
**Subset B from cohort 1: Go/no go**	6	6	0	0	0	6	6	6	0	0	6	6	0	0	0	0	0	0
**Cohort 2: qPCR analysis - aggregation assay** *	3 (2)	4 (3)	6 (3)	6 (3)	6 (3)	6 (3)	6 (3)	6 (3)	6 (3)	6 (3)	6 (3)	6 (3)	0	0	0	0	0	0

* Half of the cortex and the striatal tissues collected from each animal were used for real time qPCR analysis.

The other half striatum and cortex were used in the aggregation assay. The number in brackets indicates the sample size used for in the aggregation assay when it was different from the one used in qPCR studies.HOM; zQ175 HOM mice, HET: zQ175 HET.

One-two weeks after animals' arrival to PGI, animals were injected with sterile transponders (T-IS 8010 FDX-B, Datamars SA) under 2% isoflurane inhalation anesthesia.

#### 6.1 PhenoCube System

PhenoCube experiments were conducted using modified IntelliCage units (New Behavior AG), each with a camera mounted on top of the cage allowing for computer vision analysis. Each cage consisted of 4 corners with an area separated from the main arena, containing nosepokes with respective water bottles. In the central area, there were three types of climbing structures (two rods, a cubic central object and a three step staircase) providing enriched topography all within a rectangular housing unit. The corners were freely accessible through tunnels, which also serve as radio frequency identification (RFID) chip readers. Within each corner were two small recessed openings which allowed access to two water bottles situated in each corner. Access to the bottles was controlled by retractable doors controlled by a computer. Infra-red sensors detected nosepokes. While water was only available within the corners (with free or conditional access depending on the experimental protocol), food (14 mg dustless precision pellets, BioServ, NJ) was provided *ad libitum* on the cage floor. Mice were maintained on a 12:12 light/dark cycle, with red light during the dark cycle, allowing video capture (sample size provided in Table 1 and Table 2). The light intensity under red light was recorded at 7 lux using a photographic bandpass filter (LDP LLC, NJ) that eliminates long wavelength light frequencies not visible to mice [25]. Computer Vision analysis of the videos taken during the testing sessions tracked the movement and location of the mice in each cage generating data which included total distance travelled by the mice, total time immobile, locomotion, clustering, as well as rearing and climbing.

Following a period of water deprivation (16 hrs), mice were tested in the PhenoCube for 72 hrs sessions, initially under the Habituation protocol (6 hrs) and the remaining time under the Alternation protocol. Mice were removed from the experiment if the mouse did not lick and data from these mice were excluded from analysis. All R6/2 mice and corresponding control mice received supplemental HydroGel(ClearH20, ME) during the testing sessions at 16 weeks of age to ensure proper hydration.

Habituation: In this phase water was freely available in all four corners (Figure S1).

Alternation: During this phase subjects were required to alternate visits between two of the four corners (active corners) in order to gain access to water. Each mouse was assigned two adjacent active corners at random. Only one of the two nosepokes was activated in each corner, such that mice had to nosepoke on the left side in order to gain access to water in the right corner, or nosepoke to the right in the left corner (see Figure S1). After a correct alternation, a nosepoke in the correct recess resulted in the door opening and allowing access to the water bottle on that side. After 8 seconds, the door gently closed, preventing further access to water within a given visit. No penalty was imposed for initially nosepoking on the incorrect side.

#### 6.2 Procedural water T-maze test

Mice were tested in T-mazes constructed of black Plexiglas (locally constructed at PsychoGenics, Inc, sample size presented in Table 1 and Table 2). Arms were 33 cm high and 10 cm wide, each arm was 49 cm long. Testing was performed in a dimly-lit (approximately 15 lux) room equipped with a video camera (mounted above the T-maze) and a computer and monitor. The monitor screen was covered with red transparent film to minimize light emission. The T-maze was filled with water at 25±1°C, rendered opaque with Tempura non-toxic white paint. At one end of the cross-piece of the ‘T’, a platform was located approximately 0.5 cm below the level of the water.

In acquisition training, mice were placed in the stem of the maze and allowed to make a choice to swim into either the right or left arm to reach the escape platform, with the platform location held constant for each mouse during acquisition. A choice was defined as entry into either the left or right arm, without necessarily reaching the escape platform. Failure to leave the stem of the T-maze was defined as ‘no-choice’. Any mouse that failed to reach the platform within the maximum trial duration (60 s) was placed directly onto the platform. Once an animal reached or was placed on the platform, the animal was allowed to remain there for 30 s. Animals received 8 trials per day, for a maximum of 12 days, with an inter-trial interval of approximately 15 min. The performance criterion was set at achieving 6 or more correct trials per day for 2 consecutive days. Up to 10 days of training were provided to achieve criterion. Once criterion was achieved animals progressed to reversal testing on an individual basis. In this phase the platform was placed in the opposite choice arm. Reversal performance was assessed for 6 days. Mice were monitored at all times when in the maze.

#### 6.3 Go/No-go

Mice were tested in mouse operant chambers (Med Associates, VT) measuring 22 cm long ×18 cm wide, with 13 cm high walls (sample size presented in Table 2). Each chamber was equipped with a nosepoke recess, which could be illuminated by a small embedded light-emitting diode (LED), located centrally on the wall opposite the food magazine. Reinforcement was provided by time-limited access to a dipper containing evaporated milk (Carnation, OH). The hardware was controlled and all events were recorded by the Med-PC IV software package.

Prior to training, mice were food restricted and individually maintained at 85% of their free feeding body weights by providing them with pre-weighed rations of rodent chow on a daily basis.

Initial training. Following food restriction and two days of magazine training, all animals were trained to nosepoke via a simple free operant procedure, in which nosepoking was reinforced with 4 s access to an evaporated milk reinforcer on a response-initiated fixed-interval 20 s (FI20) schedule. The illumination of the nosepoke recess and houselight (light or dark) was counterbalanced across animals in each genotype, with the lights either on or off throughout the 40 min session. No reinforcement was delivered without a nosepoke. Animals were trained to a criterion, requiring them to obtain 40 reinforcers across two consecutive sessions, after which mice received one additional session of FI20 training.

Discrimination training. Discrimination training sessions followed completion of initial instrumental training. Discrimination sessions were also 40 min in duration, presenting the animals with both potentially reinforced and unreinforced periods, indicated by the illumination state of the nosepoke recess (the houselight was not used during this phase). The light condition presented in initial training indicated the reinforced state, such that animals were required to learn to avoid responding in the novel, unreinforced stimulus condition. No other source of illumination was utilized during discrimination training. Discrimination sessions consisted of 30 min of potentially reinforced time presented pseudorandomly in blocks of 30, 60, 90, 120 or 150 s, interspersed with 10 min of unreinforced time presented pseudorandomly in blocks of 10, 20, 30 or 60 s. Nosepoking was reinforced during the potentially reinforced periods on a response-initiated VI5 schedule with 3 s of access to the milk reinforcer, whereas nosepoking during the unreinforced period had no programmed consequence.

Discrimination performance was indexed by a discrimination ratio calculated by dividing the response rate in the reinforced condition by the sum of the response rates in the reinforced and the unreinforced conditions for each mouse for each session.

### 7. Tissue collection

Cortical and striatal tissues were dissected from naïve animals from the R6/2×TG2 KO line (n = 5–7 per genotype per sex, 12 weeks of age) and from the zQ175×TG2 KO line (n = 3–6 per genotype per sex, 52 weeks of age zQ175_HOM animals were not included). Tissues were kept frozen until processing. Half of the cortex and the striatal tissues were used for real time qPCR analysis. The other half cortex from the R6/2×TG2 KO line and the cortex and striatum from the zQ175×TG2 KO line were used in the aggregation assay. For *ex-vivo* MRI studies, a second cohort of naïve mice from the R6/2×TG2 line and the mice from zQ175×TG2 KO line tested in the procedural water T-maze test were perfused at 16 and 52 weeks of age respectively (Table 1 and Table 2). In brief, mice were killed by deep anesthesia with pentobarbital and transcardially perfused with 80 ml of heparinized (2.5 units/ml) saline followed by 80 ml of ice-cold 4% PFA in 0.1M PBS. Brains within skull were postfixed in the same fixative overnight and stored in solution of 0.01% sodium azide in 0.1M PBs at 4°C. Before the *ex-vivo* T2-MRI analysis of the brains, brains were rinsed with saline and embedded in perfluoropolyether (FOMBLIN).

### 8. Quantification of transcripts

Quantification of transcripts was performed as described previously by Menalled et al, [21]. The sample size used for each cross is presented in Table 1 and Table 2. A summary of the steps followed is described below.

#### Total RNA Extraction

Total mRNA was extracted using RNeasy 96 Universal Tissue Kit for RNA isolation (Cat # 74881, Qiagen, Valencia, CA). Total RNA bound to column membranes was then treated with RNase-Free DNase set (Cat # 79254, Qiagen, Valencia, CA), followed by washing steps with RW1 and RPE buffers (provided with RNeasy 96 Universal Tissue Kit). RNA was eluted with RNase-Free water.

#### Total RNA Quantification and Reverse Transcription

To investigate the integrity of the RNA, 2 µL of total RNA was run in 1% agarose gel. RNA was quantified using Quan-iT RiboGreen RNA Kit (Cat # R11490, Invitrogen, Carlsbad, CA) and analyzed with the SpectraMax Gemini XPS fluorescent plate reader (Molecular Devices, Sunnyvale, CA). Following the protocol previously described [21], one microgram of total RNA was reverse transcribed into cDNA.

#### Quantitative PCR (qPCR)

Five microliters of the diluted cDNA were amplified with 12.5 µL 2× FastStart Universal Probe Master Rox (Cat # 04914058001, Roche Applied Science, Indianapolis, IN), 0.5 µL Universal Probe Library Probe (Roche Applied Science, Indianapolis, IN), 200 nM of gene specific primer- HPLC purified (Sigma-Aldrich, St. Louis, MO) in 25 µL reaction volume. The reactions were run on the ABI 7900HT Sequence Detection System (Applied Biosystems, Foster City, CA). qCPR conditions were 95°C for 10 min for activation of FastStart Taq DNA Polymerase followed by 40 cycles of 95°C for 15 seconds and 60°C for 1 minute. For primers and Universal Probe Library used for qPCR refer to Table 3).

**Table 3:** Quantitative Polymerase Chain Reaction (qPCR) information.

Mouse Gene ID	5′ Primer Sequence	3′ Primer Sequence	Universal Probe Library #
***Bdnf II***	CCGGAGAGCAGAGTCCATT	CTACCACCTCGGACAAATCC	21
***Bdnf IV***	GCTGCCTTGATGTTTACTTTGA	AAGGATGGTCATCACTCTTCTCA	31
***Bdnf IX***	GCCTTTGGAGCCTCCTCTAC	GCGGCATCCAGGTAATTTT	67
***Cnr1***	GGGCAAATTTCCTTGTAGCA	GGCTCAACGTGACTGAGAAA	79
***Drd1a***	AGGTTGAGCAGGACATACGC	TGGCTACGGGGATGTAAAAG	88
***Drd2***	TGAACAGGCGGAGAATGG	CTGGTGCTTGACAGCATCTC	17
***Darpp32***	CCACCCAAAGTCGAAGAGAC	GCTAATGGTCTGCAGGTGCT	98
***Pde10A***	GAAGGCTGACCGAGTGTTTC	GGGATGGAGAGAAAGATAGGC	45
***Glt1***	GGTCATCTTGGATGGAGGTC	ATACTGGCTGCACCAATGC	83
***Htt***	TCTCATCAACCACACTGACCA	GGGGGTTAAGTGCTTCGTG	77
***Atp5b***	GGCACAATGCAGGAAAGG	TCAGCAGGCACATAGATAGCC	77
***Gadph***	CAATGTGTCCGTCGTGGATCT	GTCCTCAGTGTAGCCCAAGATG	N/A
***Eif4A2***	GCCAGGGACTTCACAGTTTC	TTCCCTCATGATGACATCTCTTT	93
***Ubc***	GACCAGCAGAGGCTGATCTT	CCTCTGAGGCAGAAGGACTAA	11

#### qPCR Data Analysis

cDNAs from multiple reverse transcription reactions were pooled together and used to create qPCR standard curves and also served as a calibrator to normalize plate to plate variations. To generate the standard curve, pooled cDNA was serially diluted from 1∶5 to 1∶1000 in RNase-free water and assayed in triplicate in each qPCR assay. The Ct values, number of cycles required for the PCR amplicon detection to reach threshold, were plotted against the logarithm value of dilution samples and a linear trend line was obtained for each gene. Each sample cDNA (diluted 1∶10) was assayed in triplicates and the Ct values averaged. Values which lie greater than 0.5 standard deviation of the average were discarded. Relative quantity of the PCR product (relative to the calibrator), the geometric means for the 3 housekeeping genes and the relative levels of the target genes were calculated as previously described [21]. *Atp5b*, *Eif4a2* and *Ubc* were used as housekeeping genes for the striatal analysis in the R6/2×TG2 KO study. *Atp5b*, *Eif4a2* and *Gadph* were used in the cortical analysis in the R6/2 CAG×TG2 KO study and in the striatal analysis of the zQ175×TG2 KO study. The relative level of the target gene was then normalized to age- and sex-matched wild type control animals.

### 9. Tissue preparation and quantitative western blotting

Frozen tissues were homogenized in ice cold modified RIPA buffer (50 mM Tris HCl, pH 7.5, 1% IGEPAL CA630, 0.25% SDS, 150 mM NaCl, 1 mM EDTA, 1 mM NaF, 100 mM activated Na2VO3 and Roche Applied Science's Complete Protease Inhibitor cocktail) using 500 µL per 100 mg of tissue with TissueLyser (Qiagen, Valencia, CA) and 5 mm stainless steel beads (Cat # 69989, Qiagen, Valencia, CA). Once tissues were disrupted, samples were allowed to incubate on ice for 30 min. The homogenates were then centrifuged at 10,000RPM (Eppendorf 5417R centrifuge) for 30 min at 4°C. The protein content was determined using BioRad's DC Assay Kit (Cat # 5000111, Bio-Rad, Hercules, CA).

Protein samples were denatured in Laemmli buffer (Cat # 1610737, Bio-Rad, Hercules, CA)/2-Mercapthoethanol (Cat # M3148, Sigma-Aldrich, St. Louis, MO) for 5 min at 95°C for TG2 and housekeeping proteins. Ten micrograms of denatured protein samples were separated by 4–20% SDS-PAGE Criterion Gels (Cat # 3450034, Bio-Rad, Hercules, CA). After electrophoresis, proteins were transferred from gel to Hybond-LFP PVDF membranes (Cat # RPN303LFP, GE Healthcare Bioscience, Piscataway, NJ) by electroblotting. The protein-PVDF membranes were rinsed briefly in 1× Tris-buffered saline with 0.1% Tween 20 (TBST). Non-specific binding was blocked with 5% dried milk in TBST for 1 hr. After a brief rinse in TBST, the blots were probed with primary antibody prepared with 1% dried milk in TBST for 1 h at RT. Protein-PVDF blots were washed 1×15 min followed by 3×5 min washes with TBST. Protein-PVDF blots were then incubated with secondary antibody prepared with 1% dried milk in TBST (see Table 4 for dilution) for 1 h at RT. Protein-PVDF blots were washed 1×15 min followed by 1×5 min.

**Table 4:** Quantitative Western Blots Antibody information.

Mouse Gene ID	Vendor	Catalog #	Host	Antibody Dilution
**TG2**	Cell Signaling Technology	3557S	rabbit	1∶500
**ATP5B**	Abcam	ab14730	mouse	1∶10000
**EIF4A2**	Abcam	ab31218	rabbit	1∶10000
**GAPDH**	Cell Signaling Technology	2118	rabbit	1∶10000
**Anti-Rabbit IgG conjugated to HRP**	Cell Signaling Technology	7074	Goat	1∶1000
**Anti-Mouse IgG conjugated to HRP**	Cell Signaling Technology	7076	Goat	1∶1000

Antibody binding was detected using ECL Plus Western Detection Kit (Cat # RPN2133, GE Healthcare Bioscience, Piscataway, NJ) with a Typhoon 9410 scanner (GE Healthcare Bioscience, Piscataway, NJ) using 457 nm blue laser for excitation and 520 nm emission filter at 400 V.

#### qWestern data analysis

Total protein from whole brain of C57BL6 mice was isolated and denatured at specific concentrations as described above. These denatured protein aliquots (of the same protein amount as target protein) served as calibrator to normalized the gel to gel variations.

The scanned images from the Typhoon were analyzed with ImageQuantTL software version 7.0 (GE Healthcare Bioscience, Piscataway, NJ), band intensities were determined using the rolling ball methods, file with band intensities was exported to EXCEL for further analysis. Within each image all the raw data values of the band intensities were corrected with band intensity from calibrator, this value was referred as corrected relative quantity, calculation as follows:
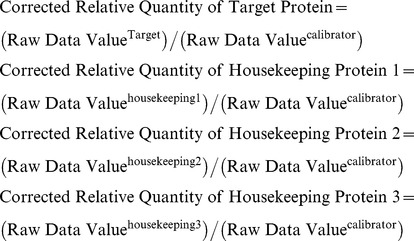
Geometric mean for the three housekeeping protein was calculated as follows:
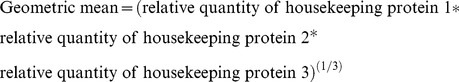
Relative level of target protein (TG2 and endogenous HTT) was calculated as follows:
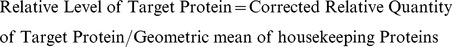
Relative level of target gene was then normalized to that of wild type (gender combined).

### 10. Measurement of polyQ aggregates by Seprion ligand ELISA

A 2.5% lysate was prepared with the dissected cerebral cortex from 12 week-old R6/2×TG2 KO and 52- week-old zQ175×TG2 KO (Table 1 and Table 2) in ice-cold RIPA buffer by ribolysing in lysis matrix tubes 2×30 sec at RT (Lysing matrix D; MP Biomedicals, Solon, OH) and transferring to ice for 3–5 min to cool before the last 30 sec ribolysing. Lysates were centrifuged at 13,000 rpm for 2 min in a microfuge before they were transferred to a fresh tube on ice. Samples either used immediately or frozen on dry ice and stored at −80°C and used within 24 h.

Homogenate (15 µl) was mixed with 3 µl 10% SDS followed by 62 µl water and made up to 100 µl with 20 µl of 5× capture buffer (Microsens Biotechnologies, UK). This was transferred to the well of a Seprion ligand-coated ELISA plate (SEP1-96-01) and incubated for 1 h at 23°C. The lysate was removed and the well was washed 5× in PBS-T (PBS; 0.1% Tween) and 100 ml S830 primary antibody (diluted 1∶2000 in conjugate buffer (150 mM NaCl; 4% BSA (98% electrophoretic grade); 1% dried milk; 0.1% Tween 20 in PBS) was added and incubated with shaking for 1 h at 23°C. The washing step was repeated and 100 µl horse radish peroxidase (HRP)-conjugated rabbit anti-goat secondary antibody (DAKO, Denmark) (1∶2000 in conjugation buffer) was added and incubated for 45 min at 23°C. The washing step was repeated and 100 µl of TMB substrate (SerTec) (warmed to 23°C) was added and incubated in the dark at RT for 30 min. Reactions were terminated by the addition of 100 µl 0.5 M HCl and the absorption at 450 nm was measured using a plate reader (Biorad, UK).

### 11. *Ex vivo* T2-MRI

MRI acquisitions from the brains collected from animals from the R6/2×TG2 KO cross (Table 1) were performed using a horizontal 7 T magnet with bore size 200 mm (Bruker Biospin GmbH, Karlsruhe, Germany) equipped with Bruker BGS12-S gradient set (max. gradient strength 760 mT/m, bore 120 mm) interfaced to a Bruker PharmaScan console (Bruker Biospin GmbH, Karlsruhe, Germany) using a volume coil for transmission and surface coil for receiving (Bruker Biospin GmbH, Karlsruhe, Germany). T2-weighted MRI acquisitions from the brains collected from the animals from the zQ175×TG2 KO cross (Table 2), were performed with the use of a horizontal 7 T magnet with inner bore diameter of 160 mm (Magnex Scientific Ltd., Oxford, UK) equipped with actively shielded Magnex gradient set (max. gradient strength 400 mT/m, bore 100 mm) interfaced to a Varian DirectDrive console. Linear RF volume-coil was used for transmission and surface phased array coil for receiving (Rapid Biomedical GmbH, Rimpar, Germany).

In all cases, for ex vivo determination of total brain, striatal and cortical volumes, T2-weighted continuous multi-slice images covering the whole brain (number of slices, 21) were acquired using fast spin-echo sequence with TR = 4500 ms, echo train length ETL = 4, effective TE = 37.7 ms, matrix size of 512×256 (zeropadded to 512×512), FOV of 30*30 mm2, and a slice thickness of 0.6 mm, 4 averages. The acquired coronal images were analyzed for total brain, striatal and cortical volumes using an analysis program ran under MatLab [22].

### 12. Statistical analysis

Body weight, PhenoCube and Go/No go test data were evaluated using repeated measures analysis carried out with SAS (SAS Institute Inc.) using Mixed Effect Models, based on the likelihood estimation. The models were fitted using the procedure PROC MIXED [26]. Main effects of HD Genotype, TG2 Genotype, sex as well as age, day/night cycle, light/dark training condition, testing day (as appropriate) were evaluated and their interactions were considered in all the models. Significant interactions followed up with simple main effects.

In Go/No go the mean number of days to reach criterion in initial training, as well as the mean of the response rates from the final four discrimination sessions were similarly analyzed, but without the repeated measure of test day.

T-maze test. Data for acquisition were analyzed using Statview software (SAS Institute Inc.) with two-way ANOVA with the factors HD Genotype and TG2 Genotype. In addition, the proportion of mice acquiring the task each day was analyzed via Kaplan-Meier analysis. For reversal testing, data were analyzed via mixed two-way repeated-measures ANOVA with the between-subject factors HD Genotype and TG2 Genotype and Days as repeated measure.

Survival data was statistically analyzed via Kaplan-Meier analysis.

Protein expression, gene expression, and ex vivo T2-MRI data were evaluated using SAS Proc Mixed with no repeated statement and significant interactions followed up with simple main effects.

In all cases an effect was considered significant if *p*<0.05.

All graphs, but body weight, represent data from male and female animals combined. Data are expressed as mean ± S.E.M.

## Results

### 1. Characterization of transglutaminase 2 protein levels in R6/2×TG2 knockout line

**Figure 1:**
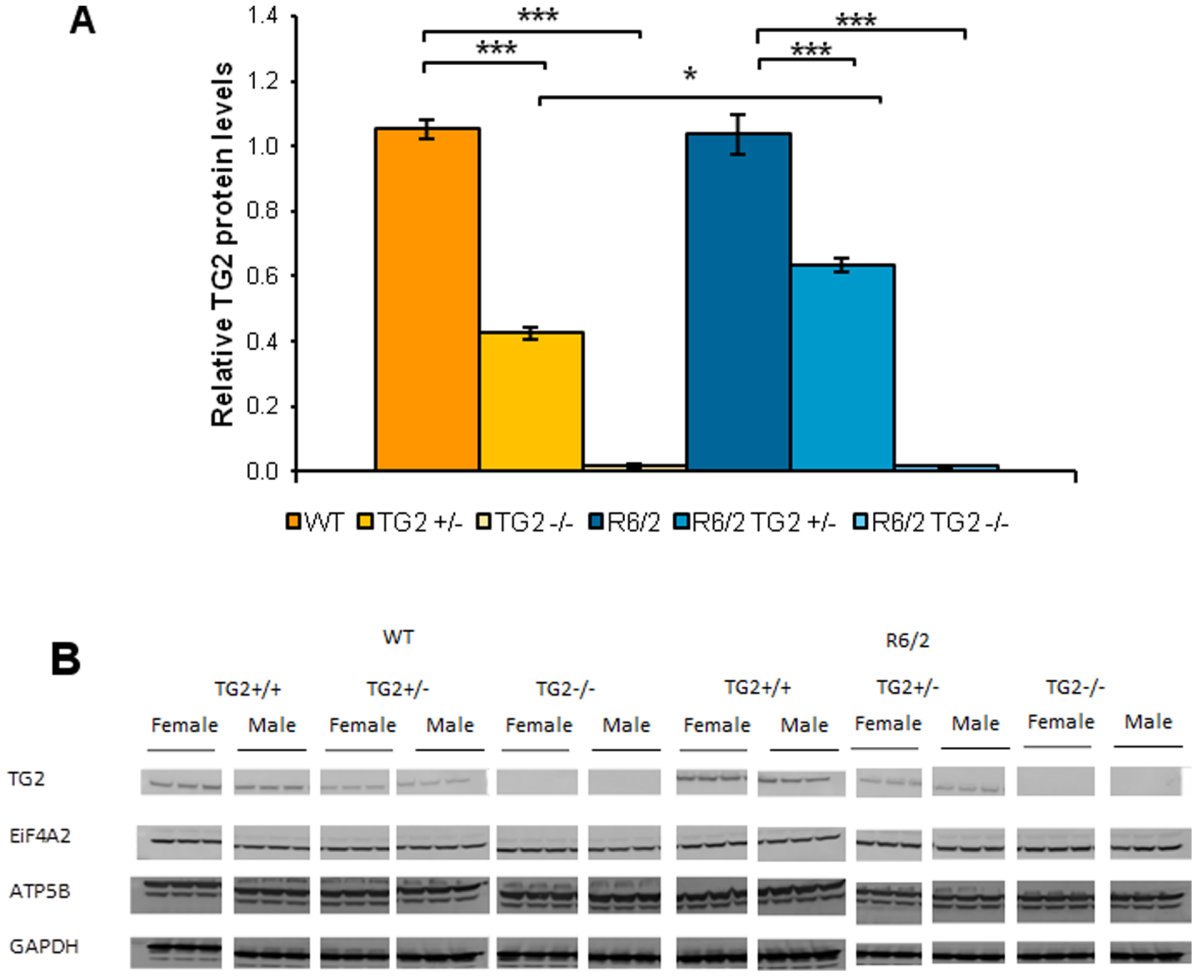
Transglutaminase 2 protein levels in R6/2×TG2 KO line. A. TG2 expression levels in the striatum of 12 week old animals from the R6/2×TG2 KO line (n = 11–12 per genotype). *p<0.05,***p<0.0001. See materials and methods' section for details regarding the calculation of TG2 protein expression levels. B. Westen blotting examples of striatal lysates of animals from the R6/2×TG2 KO line probed with antibodies that recognize TG2 and the housekeeping proteins.

In order to confirm that the TG2 expression levels were reduced both in the heterozygous (TG2+/−) and knockout (TG2−/−) mice, we performed western blotting on striatal lysates of 12-week-old mice from these crosses (Figure 1). No significant differences in the TG2 protein levels were detected between the WT and R6/2 mice. In the heterozygous TG2 animals, the protein level was approximately 50% of the wild type level, with a slightly lower protein level observed in the WTs compared to R6/2 mice (HD Genotype×TG2 Genotype interaction: F_(2,59)_ = 8.51, *p*<0.001, simple main effects and post-hoc tests *ps*<0.05). As expected no expression of TG2 protein was detected in the knockout animals (simple main effects and post hocs, *ps*<0.0001).

### 2. TG2 depletion does not alter the body weight loss phenotype of R6/2 and zQ175 KI mice

#### R6/2×TG2 KO line

**Figure 2:**
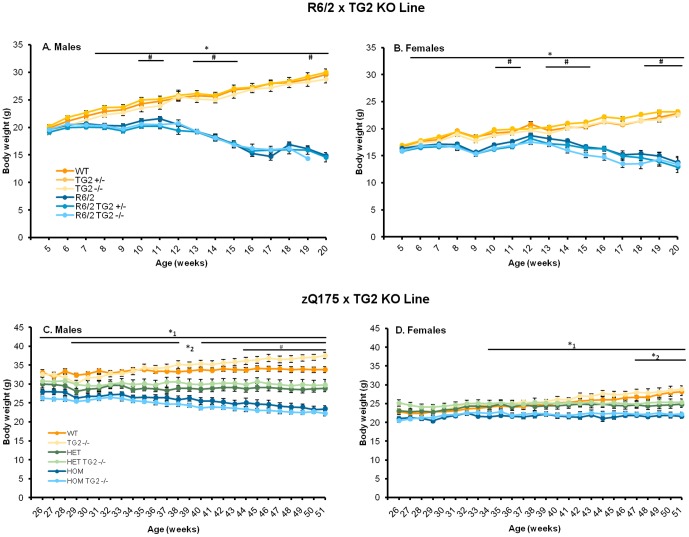
Body weight curves. A–B. Mean body weights of mice from the R6/2×TG2 KO line. A. Male mice (n = 14–16 per genotype). B. Female mice (n = 13–16 per genotype). C–D Mean body weights of mice from the zQ175×TG2 KO line. A. Male mice (n = 5–6 per genotype). B. Female mice (n = 6–7 per genotype). *Significant differences R6/2 vs WT,*^1^significant differences HOM vs WT, *^2^significant differences HET vs. WT, #significant TG2 genotype differences. WT: wild-type, TG2+/−: heterozygous TG2 knockout, TG2−/−: homozygous TG2 knockout, HET: zQ175 HET HOM: zQ175 HOM.

From the various sublines of R6/2 mice, we selected the R6/2 CAG 240 which while presenting robust deficits, offers a wider therapeutic window of intervention compared to the R6/2 CAG 110 or R6/2 CAG 160 lines [27]–[29] (manuscript in preparation). As expected from previous findings obtained from R6/2 animals carrying approximately 240 CAGs (manuscript in preparation, [27], [28]), R6/2 mice were progressively lighter than WT controls from 6 and 7 weeks of age, for males and females respectively, although the effect was more pronounced in the males (Figure 2A–B; HD Genotype×Sex×Age interaction: F_(15, 2173)_ = 11.02, *p*<0.0001; simple main effects: *ps*<0.05). Total absence of TG2 protein resulted in a reduction in body weight, independent of R6/2 genotype or sex, which reached significance at 10, 11, 14–16 and 18–20 weeks of age, (TG2 Genotype×Age interaction: F_(15,2173)_ = 98.52, *p*<0.0001; simple main effects and post hocs for ages: *ps*<0.05).

#### zQ175×TG2 KO line

Consistent with the results in the R6/2 transgenic line, the zQ175 HET and, particularly, the zQ175 HOM were progressively lighter than the corresponding WT controls, with the effect being more pronounced in the males (Figure 2C–D, males and females, respectively). The effect in the males also reached significance at younger ages (29 and 26 weeks of age, for HET and HOM mice, respectively) than in the females (48 and 34 weeks of age, for zQ175 HET and HOM mice, respectively;; HD Genotype×Sex×Age: F_(50,1475)_ = 2.24, *p*<0.0001; simple main effects: *p*<0.05). The TG2 KO male mice presented a significantly increased body weight when compared to WT animals from 44 weeks of age (HD Genotype×TG2 Genotype×Sex×Age interaction: F_(50,1475)_ = 3.28, *p*<0.0001; simple main effects and post-hocs, *ps*<0.05).

### 3. TG2 depletion does not impact the survival of HD model mice

#### R6/2×TG2 KO line

WT mice had a normal life span during the period examined, regardless of the level of TG2 protein. The reduced lifespan characteristic of the R6/2 was not improved by the TG2 protein level (Mean survival ± S.E.M.: 18.85±0.43 weeks, 18.93±0.40 weeks and 17.42±0.45 weeks for the R6/2, R6/2 TG2+/−: and R6/2 TG2−/− respectively; Median survival: 17.71, 19.26 and 16.86 weeks for the R6/2, R6/2 TG2+/− and R6/2 TG2−/− respectively; HD Genotype log-rank test, *p*<0.0001; TG2 Genotype log-rank test, *p*>0.8).

#### zQ175×TG2 KO line

HOM and HET animals from the zQ175×TG2 cross displayed equivalent lifespan to the controls during the period examined (data not shown).

### 4. TG2 depletion does not improve the deficits detected in the R6/2 and zQ175 KI mice in PhenoCube System

#### R6/2×TG2 KO line

**Figure 3:**
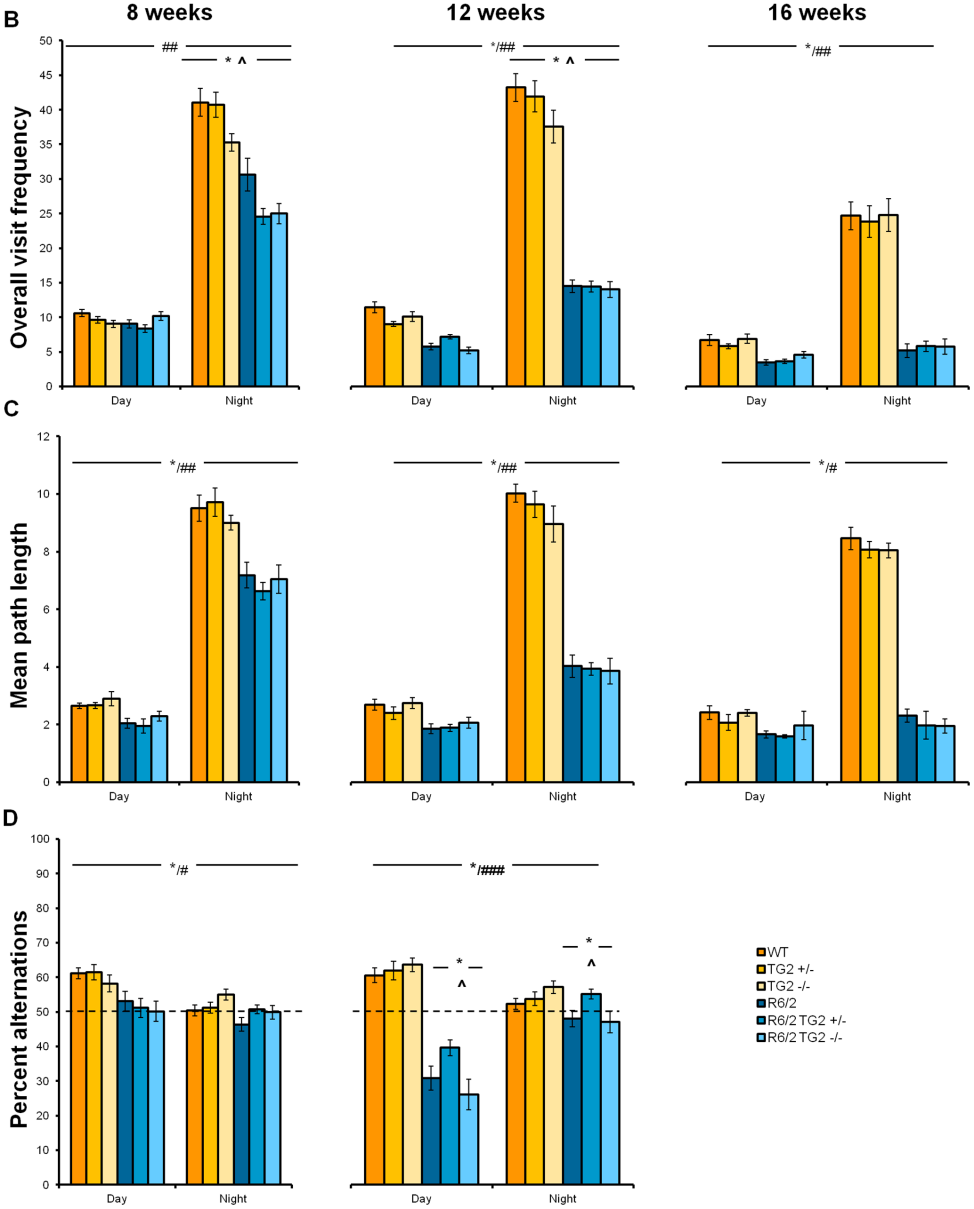
Behavioral data captured in the PhenoCube system as a function of genotype, age and light cycle phase in animals from the R6/2×TG2 KO line. A. Overall visit frequency. B. Mean path length. C. Percent alternations (Data for this measure were not collected at 16 weeks of age since R6/2 mice were not tested in this protocol due to reduced licking). *Significant HD genotype differences within each light phase, at each age; #: significant differences due to light phase in the diurnal cycle in the WT mice; ##significant differences due to light phase in the cycle for each age independently of genotype; ###significant differences due to light phase in the diurnal cycle in the R6/2 mice; ∧significant TG2 genotype differences. WT: wild-type, TG2+/−: heterozygous TG2 knockout, TG2−/−: homozygous TG2 knockout.

Overall visit frequency: R6/2 showed a progressive age-related decrease in activity when compared to WTs in all the phases of the diurnal cycle with the exception of 8 week old mice during the light phase (Figure 3A; HD Genotype×Cycle×Age interaction: F_(2,319)_ = 16.50, p<0.0001; simple main effects and post hocs, ps<0.05). Both partial or complete reduction of the TG2 protein expression produced a decrease in overall visit frequency during the dark phase regardless of the status of the R6/2 transgene, mainly driven by male mice at 8 and 12 weeks of age (not shown, TG2 Genotype×Cycle interaction: F_(2, 177)_ = 4.24, p<0.05, simple main effect and post hocs, ps<0.05; TG2 Genotype×Age×Sex interaction: F_(4,301)_ = 3.3, p<0.05; simple main effects and post hocs, ps<0.05). As expected, mice displayed a marked increase in corner visit frequency during the dark versus light periods of the diurnal cycle irrespectively of the genotype (Cycle main effect: F_(1,133)_ = 433.07, p<0.0001).

Mean path length: As previously observed [24], a progressive decrease in locomotion activity in the R6/2 mice was detected when compared to the WT animals (Figure 3B; HD Genotype×Age interaction: F_(2,36)_ = 24.13, p<0.0001; simple main effect and post hocs, ps<0.05). While all animals presented the expected higher levels of locomotion during the dark phase than light phase of the diurnal cycle, this was not observed in the R6/2 mice at 16 weeks of age (HD Genotype×Cycle×Age interaction: F_(2,36)_ = 31.03, p<0.0001; simple main effect and post hocs, ps<0.05). The partial or complete depletion of TG2 did not impact the animals' performance.

Percent alternations: R6/2 mice presented a decreased percent alternations between corners where water could be obtained (active corners) when compared to WT animals. This was observed both during the light and dark phases of the diurnal cycle, at 8 and 12 weeks of age, irrespectively of the TG2 expression level (Figure 3C; HD Genotype×Age×Cycle interaction: F_(1,141)_ = 34.36, p<0.0001; simple main effects and post hocs, ps<0.05). As expected, during the light phase WT mice presented a higher percentage of alternations than during the dark phase, regardless of the age (simple main effects and post hocs, ps<0.05). R6/2 mice, however, presented no differences in alternations during the light phase at 8 weeks of age (simple main effects and post hocs, ps<0.05). Interestingly, the percent alternations displayed by R6/2 at 12 weeks of age were below chance during the light phase. This is in alignment with an increased overall repeat visits observed in R6/2 animals in the same cycle period and age (data not shown). Surprisingly, the partial, but not the total, depletion of the TG2 protein increased alternations in R6/2 mice at 12 weeks of age, mainly in the male mice (HD Genotype×TG2 Genotype×Age interaction: F_(2,132)_ = 3.34, p<0.05; TG2 Genotype×Sex interaction: F_(1,165)_ = 3.25, p<0.05; simple main effects and post hocs, ps<0.05). Data for this measure were not collected at 16 weeks of age since the alternation protocol was not used for R6/2 mice at this age, due to their reduced licking.

### 5. zQ175×TG2 KO line

**Figure 4:**
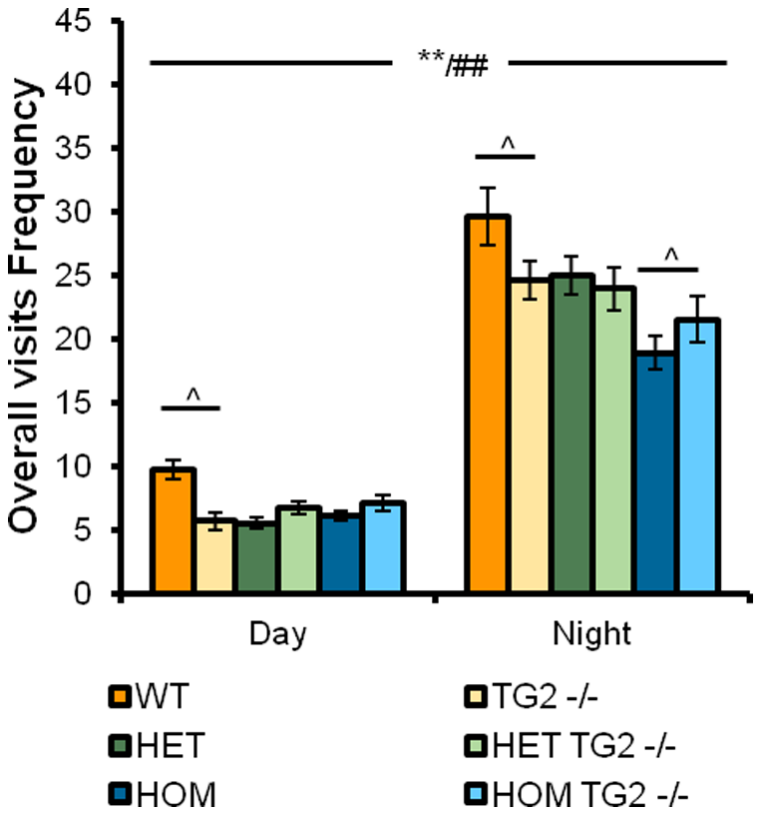
Overall visit frequency detected in the PhenoCube system as a function of genotype, age and light cycle phase in animals from the zQ175×TG2 KO line. *Significant HD genotype differences regardless of the light phase (in the mean path length the differences were detected between HET and WT animals); ##significant differences due to light phase in the cycle independently of genotype; ∧significant TG2 genotype differences (see results method for details). WT: wild-type, TG2−/−: homozygous TG2 knockout, HET: zQ175 HET, HOM: zQ175 HOM.

Overall visit frequency: Both zQ175 HOM and HET mice showed a significantly decreased visit frequency when compared to the WT mice (Figure 4; HD Genotype main effect: F_(2,110)_ = 6.72, p<0.005). The absence of the TG2 protein produced a small but significant increase of activity in HOM male mice, in the dark phase of the cycle. In both female and male WT mice, the effect was, instead, a significant decrease, observed during both phases in females but only in the dark phase in males (HD Genotype×TG2 Genotype×Sex×Cycle interaction: F_(2,16)_ = 4.11, p<0.05; simple main effects and post hocs, ps<0.05). As expected, a marked increase in corner visit frequency during the dark versus the light periods was observed in all mice regardless of the genotype and sex (Cycle main effect: F_(1,110)_ = 802.22, p<0.0001).

### 6. TG2 depletion does not improve the cognitive deficits of R6/2 and zQ175 KI mice

#### Procedural water T-maze test: R6/2×TG2 KO and zQ175×TG2 KO lines

The procedural water T-maze test allows the evaluation of cognitive abilities of animals. Mice were trained to find a submerged escape platform located in one of the arms of the T-maze without the aid of visual/light cues. Performance on this task is attributed primarily to striatal function.

#### R6/2×TG2 KO line: 14 weeks of age

**Figure 5:**
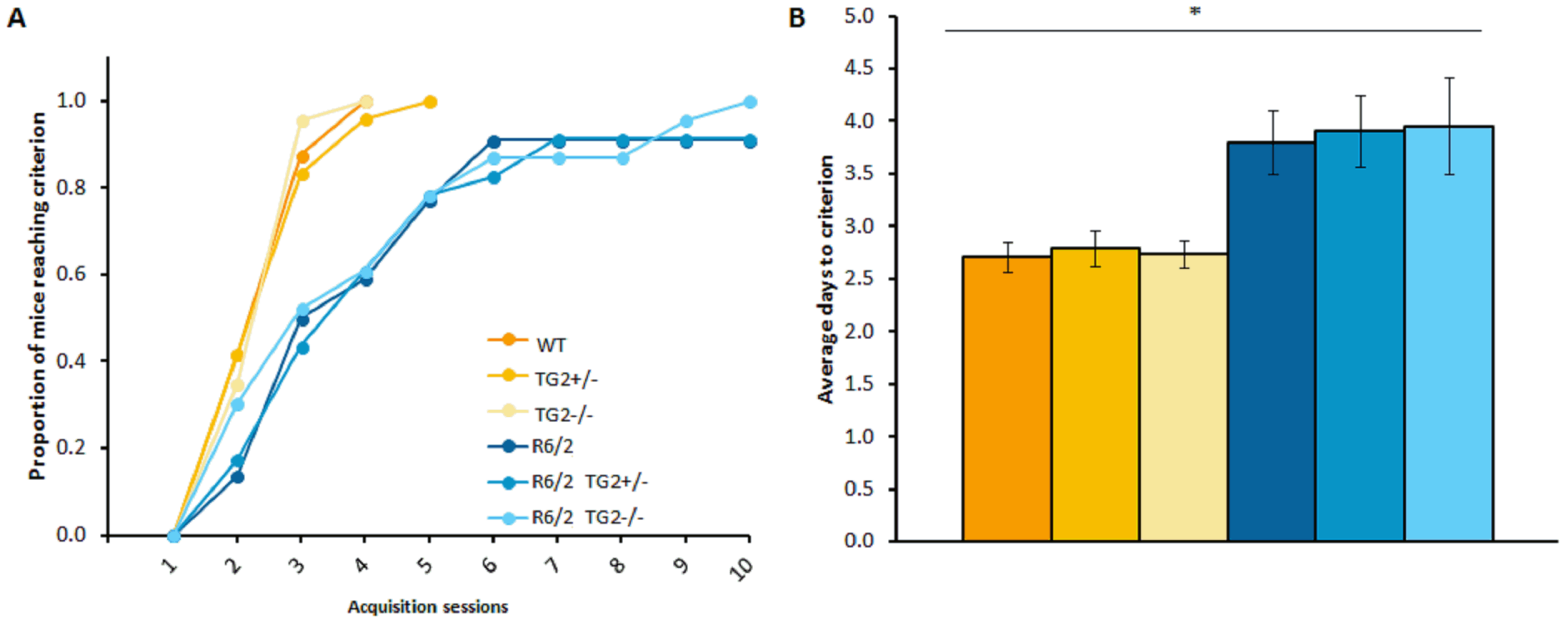
Acquisition of the procedural T-maze task (n = 20–24 per genotype). A. Proportion of mice acquiring the task on each test day. B. Average (±SEM) number of days to acquire the task (only animals that fulfill the acquisition criterion within 10 training days were included). *Significant HD genotype effect. WT: wild-type, TG2+/−: heterozygous TG2 knockout, TG2−/−: homozygous TG2 knockout.

Acquisition phase. R6/2 mice were impaired in the acquisition of the task relative to WT animals. A significantly lower proportion of R6/2 mice reached the acquisition performance criterion with successive days of training compared to WT mice (Figure 5A; Log Rank test, p<0.05). Consequently, R6/2 required a significantly higher average number of days to reach the performance criterion relative to WT mice (Figure 5B; HD Genotype main effect: F_(1,123)_ = 25.74, p<0.0001). The partial or complete depletion of TG2 protein had no impact on these features.

**Figure 6:**
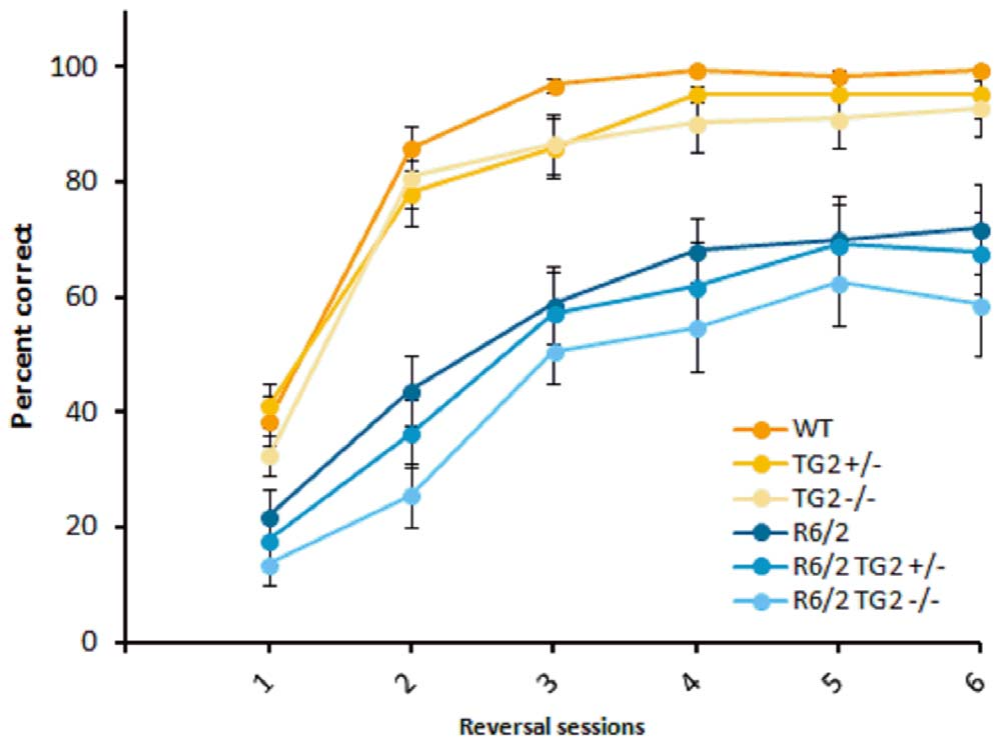
Reversal phase of the procedural T–maze task (platform location switched; n = 19–24 per genotype). The graph shows the mean (±SEM) percent correct for each group, on each test day. WT: wild-type, TG2+/−: heterozygous TG2 knockout, TG2−/−: homozygous TG2 knockout.

Reversal phase. Animals progressed to reversal testing on an individual basis once criterion was achieved in the acquisition phase. From the R6/2 TG2+/− mice, 2 mice did not reach criterion even after 10 days of training and therefore were not evaluated under the reversal conditions. Two out of 21 R6/2 TG2+/− mice and 3 out of 23 R6/2 TG2−/− mice that achieved criterion failed to complete 6 days of reversal testing due to requiring more than 6 days to meet criterion in the acquisition phase; consequently, their data were excluded from the reversal phase. Performance in the reversal task phase improved over days of testing regardless of genotype although, as expected, R6/2 mice exhibited lower accuracy relative to WT mice as testing progressed, with R6/2 mice performing worse on each day of testing (Figure 6; HD Genotype×Session interaction: F_(5,590)_ = 8.12, p<0.0001). Notably, the complete absence of TG2 protein further impaired the performance of the R6/2 female mice (HD Genotype xTG2 Genotype×Sex interaction: F_(2,118)_ = 4.90, p<0.05: simple main effects and post hocs, p<0.05). No effect of the partial or complete depletion of the TG2 protein was detected in the male group.

#### zQ175×TG2 KO line: 28–29 weeks of age

**Figure 7:**
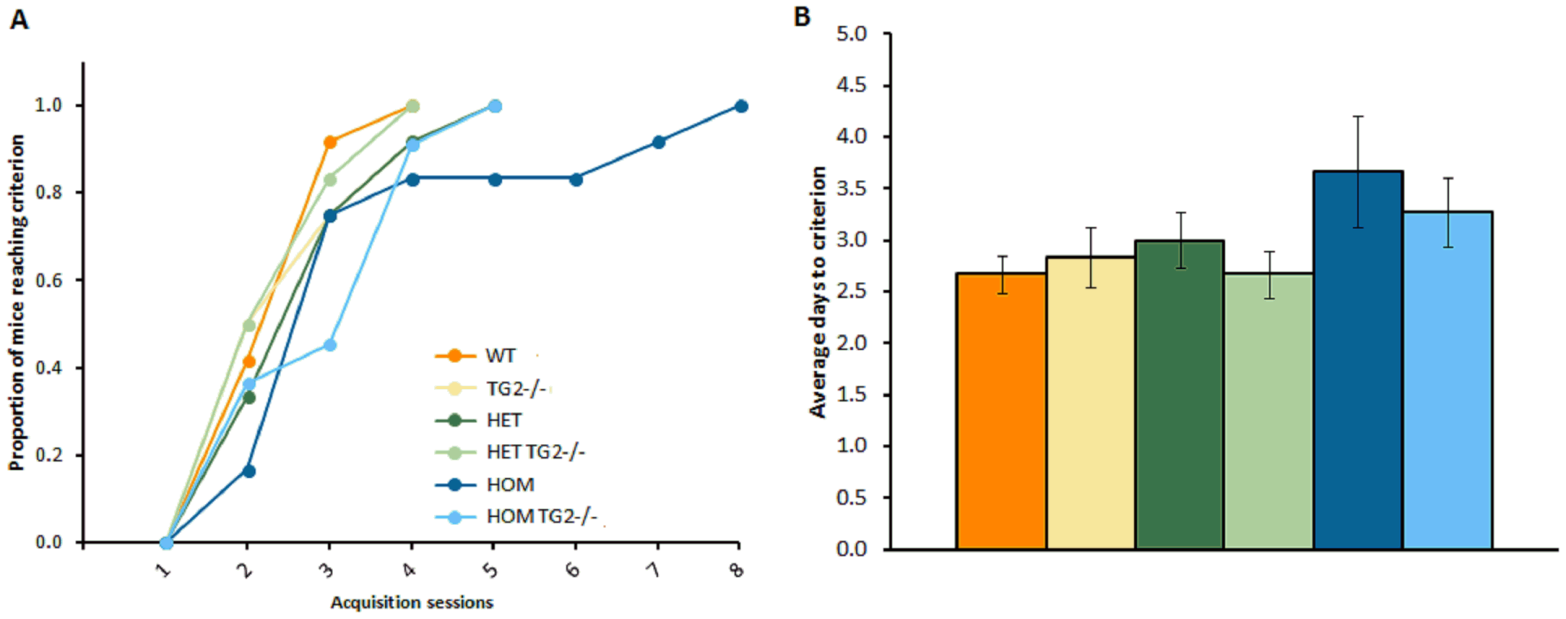
Acquisition of the procedural T-maze task in the zQ175×TG2 KO animals (n = 11–12, per genotype). A. Proportion of mice acquiring the task on each test day. B. Average (±SEM) number of days to acquire the task. WT: wild-type, TG2+/−: heterozygous TG2 knockout, TG2−/−: homozygous TG2 knockout, HET: zQ175 HET, HOM: zQ175 HOM.

Acquisition phase. Although the zQ175 HOM required more days to reach criterion when compared to WT, this difference did not reach significance (Figure 7A–B). Consistent with the results in R6/2 mice, the complete ablation of TG2 did not impact the performance of the animals in this phase.

**Figure 8:**
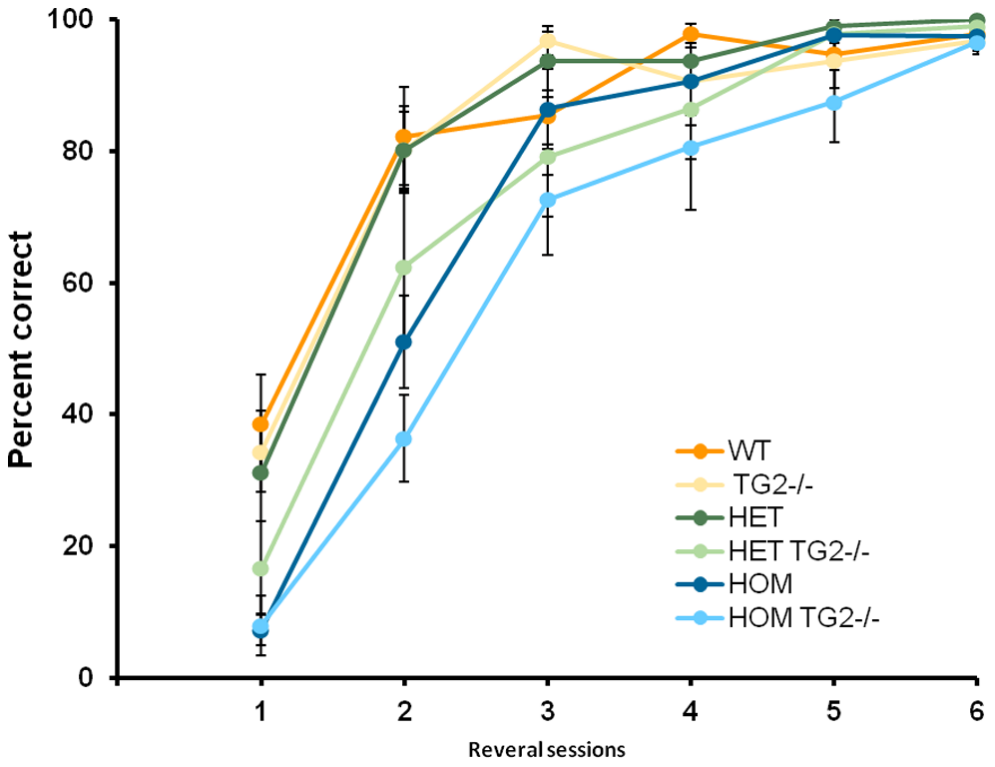
Reversal phase of the procedural T–maze task (platform location switched; n = 11–12 per genotype). WT: wild-type, TG2−/−: homozygous TG2 knockout, HET: zQ175 HET, HOM: zQ175 HOM.

Reversal phase. Similar to R6/2 mice, zQ175 HOM mice exhibited impaired accuracy relative to WT, with WT mice improving in performance at a higher rate than zQ175 HOM mice (Figure 8, HD Genotype×Session interaction: F_(10,292)_ = 3.36, p<0.0001; simple main effects and post hocs, p<0.05). No deficits were detected in the performance of the HET mice. In agreement with our results in the R6/2×TG2 KO line, TG2 deletion significantly impaired performance on accuracy during reversal learning, in this line however, this effect was observed regardless of the zQ175 zygosity (TG2 Genotype main effect: F_(1,59)_ = 4.29, *p*<0.05).

#### Go/No go discrimination task – zQ175×TG2 KO line

Since no cognitive deficits were detected in the zQ175 HET mice in the procedural water T-maze test, we extensively evaluated the HET mice in other assays focusing on executive function. Using a Go/No go discrimination task, we were able to uncover important cognitive deficits in discrimination learning and response inhibition in zQ175 HET mice (manuscript in preparation). For that reason we selected this assay to evaluate if TG2 ablation was able to modify the deficits observed in the zQ175 HET mice from 32 to 34 weeks of age.

**Figure 9:**
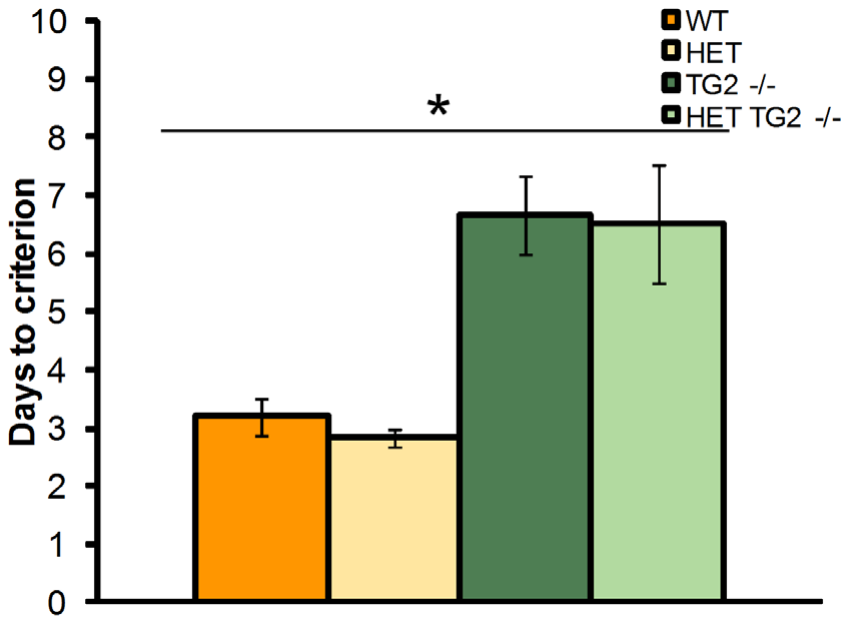
Mean number of days required to obtain 40 reinforcers across two consecutive sessions during the training phase (n = 6–12 per genotype). *Significant HD genotype effect. WT: wild-type, TG2−/−: homozygous TG2 knockout, HET: zQ175 HET.

Initial training. zQ175 HET mice required significantly more training sessions in order to achieve the response acquisition criterion of 40 reinforcers across two consecutive sessions (Figure 9; HD Genotype main effect: F_(1,31)_ = 13.40, p<0.001). The deletion of TG2 had no significant effect on this measure.

**Figure 10:**
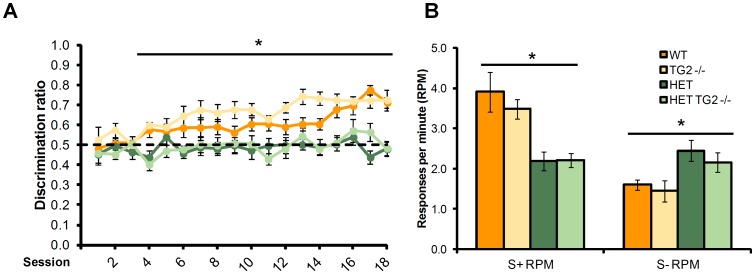
Discrimination performance (n = 6–12 per genotype). A. Discrimination ratio across the training period. B. Response rate for the reinforced (S+ RPM) and unreinforced (S- RPM) conditions averaged across the final four sessions for the zQ175×TG2 KO line. *Significant HD genotype effect. WT: wild-type, TG2−/−: homozygous TG2 knockout, HET: zQ175 HET.

Discrimination ratio. zQ175 HET mice exhibited an impairment in discrimination performance relative to WT (Figure 10A; HD Genotype main effect: F_(1,31)_ = 127.07, p<0.0001). Moreover, WT, but not zQ175 HET, mice significantly improved in their discrimination performance over days of testing (HD Genotype×Session interaction: F_(17,515)_ = 2.03, p<0.005; simple main effect, ps<0.05). Deficits in discrimination performance in the HET mice were detected from session 4 onward (simple main effect, ps<0.05). Interestingly, knockout TG2 mice performed better than TG2 wild type mice, regardless of zQ175 genotype (TG2 genotype main effect, _(1,32)_ = 6.00, p<0.05). There was also a significant interaction between light/dark condition and session, with mice trained with the stimulus light off for the reinforced condition performing better in the first session (Stimulus×Session interaction: F_(17,515)_ = 1.95, p<0.05; data not shown).

Response per minute. WT mice preferentially responded at a higher rate to the reinforced stimulus, whereas zQ175 HET mice did not exhibit differential responses rates to the reinforced and unreinforced stimulus conditions (Figure 10B; HD Genotype×Reinforcement Condition interaction: F_(1,30)_ = 47.88, *p*<0.0001; simple main effects, *ps*<0.05). Accordingly, while zQ175 HET mice responded at a lower rate than WT mice during the reinforced condition, their response rate exceeded that of the WT mice in the unreinforced condition (simple main effects, *ps*<0.05). TG2 ablation did not affect any of these deficits observed in the HET mice.

### 7. TG2 depletion does not modify the expression levels of dysregulated transcripts in brains of R6/2 and zQ175 KI mice

We examined whether the ablation of TG2 was able to ameliorate the dysregulation of genes that express key striatal neurotransmitter receptors and intracellular signalling molecules which have been found to be affected both in patients and in HD animal models (for a review see [30]). In addition, we examined whether the ablation of TG2 was able to ameliorate the decreased expression of brain-derived neurotrophic factor (*Bdnf*) which has also been found to be decreased in numerous HD mice and in human post-mortem tissue (for a review see [31]).

#### R6/2×TG2 KO line

**Figure 11:**
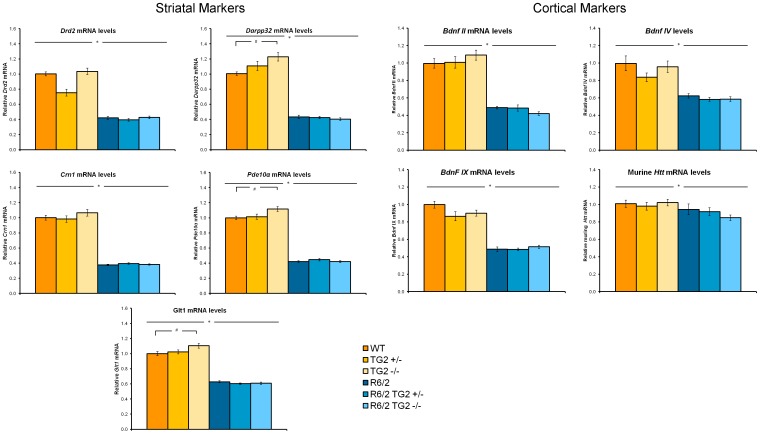
The relative striatal (left panels) and cortical (right panels) mRNA expression level of mice examined from the R6/2×TG2 KO line at 12 weeks of age (n = 12 per genotype). Relative mRNA levels are normalized to WT controls. For normalization, the geometric means of *Ubc*, *Eif4a2* an *Atp5b* were used. *Significant HD Genotype effect; #significant TG2 Genotype effect. WT: wild-type, TG2+/−: heterozygous TG2 knockout, TG2−/−: homozygous TG2 knockout.

Striatal markers. qPCR analysis revealed decreased expression of Drd2, Darpp32, Pde10a, Cnr1 and Glt1 mRNAs in the striatum of 12-week-old R6/2 mice when compared to age matched WT animals (Figure 11, left panels; HD Genotype main effects: Fs_(1,60)_>500.34, ps<0.0001). The partial or complete absence of TG2 protein did not impact the deficits in the expression levels of those striatal markers in the R6/2 mice. However, in the WT mice, the knockout of TG2 produced a small but significant increase of Darpp32, Pde10a and Glt1 mRNA levels when compared to the heterozygous and wild type TG2 animals (HD Genotype×TG2 Genotype interaction: Fs_(2,60)_>3.605, p<0.05; simple main effects and post hocs, ps<0.05).

Cortical markers. Cortical levels of the Bdnf isoforms examined, namely isoform II, IV and IX, were found to be significantly reduced in the R6/2 mice when compared to WT animals, irrespective of the TG2 protein level (Figure 11, right panels; HD Genotype main effect: Fs_(1,59)_>60.861, ps<0.0001). Cortical mRNA levels of endogenous huntingtin (Htt) were also found to be decreased in R6/2 mice compared to WT animals (HD Genotype main effect, Fs_(1,59)_>6.825, ps<0.05). The partial or total ablation of TG2 did not impact the deficits detected in the R6/2 animals in the cortical markers measured.

#### zQ175×TG2 KO line

**Figure 12:**
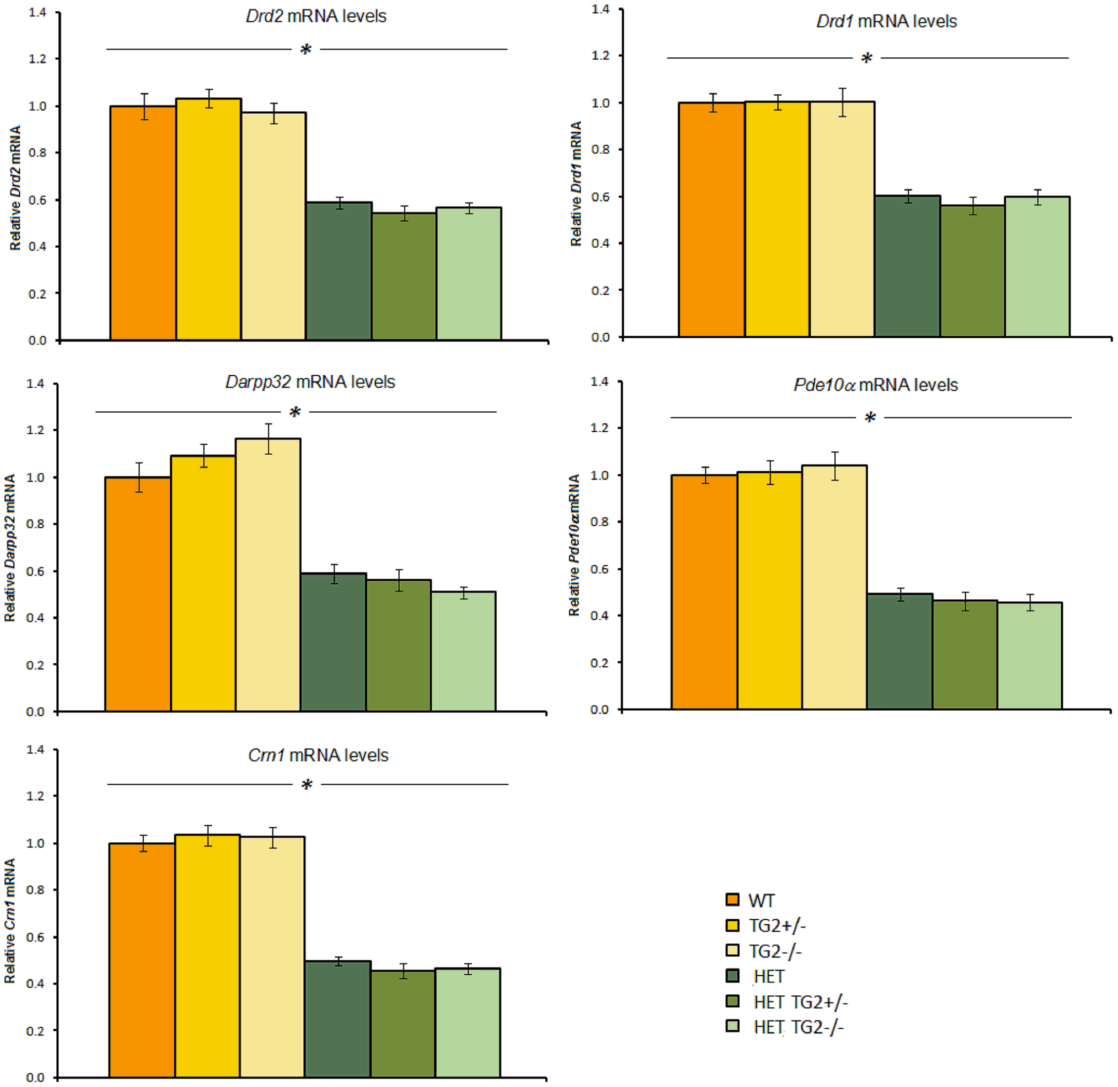
The relative striatal mRNA expression level of mice examined from the zQ175×TG2 KO line at 12 months of age (n = 7–12 per genotype). Relative mRNA levels are normalized to zQ175_WT TG2_WT controls. For normalization, the geometric means of *Ubc*, *Eif4a2* an *Atp5b* were used. *Significant HD Genotype effect. WT: wild-type, TG2+/−: heterozygous TG2 knockout, TG2−/−: homozygous TG2 knockout, HET: zQ175 HET.

Striatal markers. As expected [21], 52-week-old zQ175 HET mice presented a significant downregulation of Drd2, Darpp32, Pde10α, Cnr1 and Drd1a striatal mRNAs when compared to age matched WT animals (Figure 12; HD Genotype main effect: Fs_(1,55)_>144.62, *ps*<0.0001). The partial or complete depletion of TG2 protein did not impact the decreased expression of those transcripts.

### 8. TG2 depletion does not reduce aggregate load or attenuate brain atrophy in R6/2 and zQ175 KI mice

**Figure 13:**
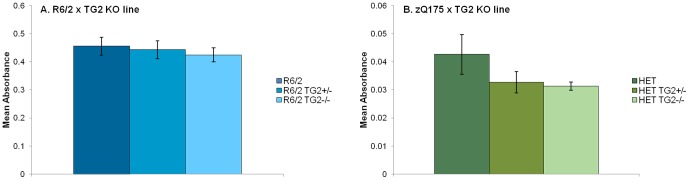
Seprion ligand quantification of aggregate load. A. Aggregate load in cortical tissues from 12-week-old R6/2×TG2 KO mice. The background readings obtained from the WT animals (n = 3 per group) were averaged and subtracted from the readings obtained from the R6/2 animals in order to remove the baseline reading. B. Aggregate load in striatal tissues from 52-week-old zQ175×TG2 KO mice. The readings obtained from the zQ175_WT animals (n = 5–7 per group) were averaged and subtracted from the readings obtained from the zQ175_HET animals in order to remove the baseline reading. WT: wild-type, TG2+/−: heterozygous TG2 knockout, TG2−/−: homozygous TG2 knockout, HET: zQ175 HET.

Using the Seprion ligand ELISA [32], we quantified the polyglutamine aggregate load in the cortex of 12-week-old animals from the R6/2×TG2 KO line and in cortex and striatum of 12-month-old heterozygous mice from the zQ175×TG2 KO line. The aggregate levels detected in the R6/2 were not impacted by the partial or total absence of TG2 protein (Figure 13A). In the zQ175 HET mice, we were unable to detect an aggregate signal in cortex at 12 months of age. The signal in striatum was comparatively low, but similar to the R6/2 results, the aggregate levels detected in the zQ175 HET were not impacted by the partial or total depletion of TG2 protein (Figure 13B).

**Figure 14:**
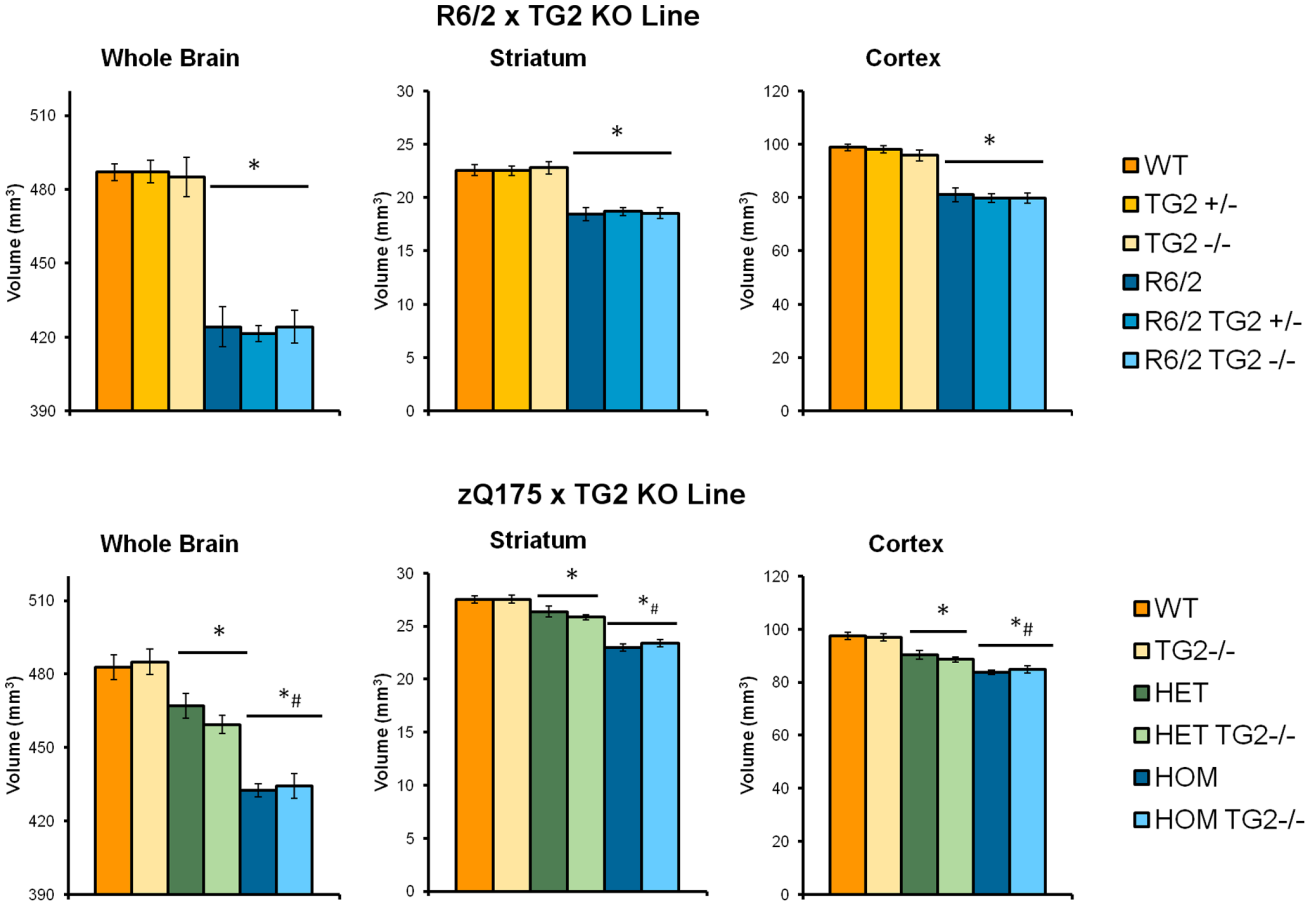
Whole brain, striatal and cortical volumes of 16-week-old R6/2×TG2 KO mice (top panels, n = 6 per genotype per sex) and of 52-weeks-old zQ175×TG2 KO mice (bottom panels, n = 6 per genotype per sex). *Significant differences compared to WT animals, #significant differences compared to HET mice. WT: wild-type, TG2+/−: heterozygous TG2 knockout, TG2−/−: homozygous TG2 knockout, HET: zQ175 HET, HOM; zQ175HOM.

Using ex vivo T2-MRI analysis, we measured whole brain, striatal and cortical volumes in 16-week-old R6/2×TG2 KO animals and 52-week-old zQ175×TG2 KO animals (Figure 14).

Total brain, striatal and cortical volumes in R6/2 mice were significantly reduced when compared to the WTs, regardless of the TG2 protein level (Figure 14, top panels; HD Genotype main effect: Fs_(1,60)_>90.578, *ps*<0.0001). Similarly, zQ175 HOM and HET mice presented significantly reduced brain volumes when compared to the WTs, regardless of the TG2 protein level (Figure 14, bottom panels; HD Genotype main effect: Fs_(2,60)_>61.13, *ps*<0.0001; post hocs, *ps*<0.0001). Also, in the zQ175 HOM mice those volumes were also significantly reduced when compared to the zQ175 HET mice (post hocs, *ps*<0.0001). Whole brain and cortical volumes of females were significantly larger than those of males (not shown, Sex main effect: F_(1,60)_>12.61, *ps*<0.001).

## Discussion

We employed a genetic deletion approach to further examine the possible role that TG2 plays in HD. A TG2 knockout line was crossed with both a transgenic and a knock-in mouse model of HD, the R6/2 and the zQ175 lines, respectively. We evaluated the resulting crosses using a behavioral testing battery under moderated level of environmental enrichment. In addition, we further examined the effect of the TG2 ablation using molecular and imaging techniques.

Surprisingly, we did not replicate the positive results previously reported by Bailey and Johnson using the R6/2 HD model [17]; they observed that the TG2 ablation extended the lifespan of R6/2 animals carrying 155–175 CAG repeats in a mixed background strain [17], [33], similar to results obtained with an R6/1 line [16] (see table S1 for a comparison of present results to the ones previously described). However, we were unable to replicate the increased lifespan when using R6/2 mice carrying 232–272 CAG repeats in a congenic C57BL/6J strain. It is well established that R6/2 mice carrying approximately 240 CAG repeats have a longer life span than those carrying approximately 150 CAG repeats [28], [29]. It is possible that the previous report of increased survival in R6/2 mice after TG2 ablation could be modulated by the CAG repeat of the model studied. Another possible explanation as to why we did not see difference in R6/2 survival after TG2 ablation may be because of the housing conditions utilized in our study, which have been shown previously to improve survival [34], [35]. Since our goal is to uncover robust targets for HD, we provided the animals with a moderate enriched environment (namely: group housing, a range of bedding, plastic bones and tunnels) as well as easy access to food and water. These husbandry conditions produce survival data that more accurately reflects mortality due to the mutation rather than malnourishment or dehydration consequence of the motor deterioration [34]. When husbandry conditions are optimized, disease pathology is better isolated allowing more stringent evaluation of potential therapies. It is under these husbandry conditions that the ablation of TG2 failed to improve the survival of R6/2 animals. Survival analysis was not performed on the TG2×zQ175 cross since the results obtained did not warrant this long term study [21].

In our hands, TG2 ablation also did not to delay the onset of, or otherwise ameliorate, the robust locomotor deficits detected by the PhenoCube system, an automated and high throughput platform that allows unbiased evaluation of animal behavior [36]. Using a constant speed rotarod protocol, Bailey and Johnson observed a delay in the onset of motor dysfunction produced after TG2 ablation, an improvement that did not translate to their ambulatory observations [17], in agreement with our results. Given the burden of cognitive decline for HD patients and their caregivers, a long term goal has been to utilize robust cognitive tests (especially those focusing on cognitive flexibility) that could be used to evaluate the effectiveness of therapies or validity of targets in mouse models. We therefore examined the ability of TG2 ablation to reverse the deficits detected in the procedural T-maze in R6/2 and zQ175 homozygous mice, as well as in the Go/No-go discrimination/response inhibition assay in zQ175 heterozygous mice, but we did not see beneficial effects. Different tests are required due to milder cognitive deficits observed in the HET mice in the T-maze at 28–29 weeks of age.

Interestingly, the TG2 ablation in R6/2 and R6/1 mice was reported to increase the number of HTT aggregates or neurons presenting aggregates, respectively [16], [17]. While this was originally unexpected, *in vitro* studies have suggested that aggregates can be formed independently of TG2 through polar zippers [37], [38]. Furthermore, TG2 knockout mice present impaired autophagy and an accumulation of ubiquitinated protein aggregates [39]. We did not detect an increase in aggregate load in either of the mouse models examined after TG2 ablation. TG2 has been associated with the presence of nuclear actin-cofilin stress rods in cells expressing mutant huntingtin [40]. While we did not monitor for these ‘cofilin rods’ in the brains of the TG2 KO crosses, the lack of amelioration of any disease phenotype in these animals suggests that TG2 activity is either not required for the formation of these rods in a physiological context, or that these rods are not relevant to disease progression in the R6/2 and zQ175 HD models. It is possible that the huntingtin aggregate burden in 12 week-old R6/2 and 52 week-old zQ175 mice prevented the detection of a minor aggregate load differences due to a ceiling effect. This seems less likely for the zQ175 mice as the Seprion signal is still relatively low at this age in the tissues examined. Alternatively, the differences with the some of the previously published studies regarding the aggregate levels may be related to the antibodies used. In the Seprion ligand-ELISA assay we used antibodies recognizing the N-terminal portion of human huntingtin protein that have been previously shown to stain HTT inclusion bodies in mouse brain tissue as well as detect oligomeric, proto-fibrillar and fibrillar aggregates using electron microscopy, atomic force microscopy (AFM) and sodium dodecyl sulphate–polyacrylamide gel electrophoresis [32], [41]. Future studies will examine the role of TG2 in regulating autophagic responses in HD by investigating levels of autophagy markers such as p62 and autophagosomes as well as specifically tracking ubiquinated forms of HTT and other proteins that are predicted to accumulate in TG2 KO mice [39].

TG2 inhibition by a novel peptide, ZDON, has been shown to normalize around 40% of the genes dysregulated in HdhQ111 immortalized striatal neurons [42]. Our data showed that TG2 ablation in the HD mouse models examined had no effect on the downregulation of expression of the group of genes examined. In agreement with Bailey and Johnson [17], we also found that TG2 ablation did not affect the histopathological endpoints examined, namely striatal, cortical and whole brain volumes.

In summary, under rigorous experimental conditions we found that the genetic ablation of TG2 does not ameliorate the disease phenotype, including physiological, molecular, neuropathological and behavioral markers in two different HD mouse models. Supporting our results, the overexpression of TG2 in R6/2 mice also did not modify the disease phenotype [43], raising additional questions about the role of TG2 activity in HD. The results of our genetic target validation work with a transglutaminase 2 null gene on the background of 2 different mouse models of HD do not support a therapeutic path aimed at TG2 inhibition for the treatment of HD.

## Supporting Information

Figure S1 Graphical depiction of cage plan and water access protocol during Habituation and Alternation phases. Shaded circles signal the armed receptacles (NP: Nosepoke).
(TIF)

Table S1 Effect of TG2 deletion on HD mouse models: comparison of present results to previously published ones.
(DOCX)
