# Supplementary figures and images for: Genetic Deletion of Transglutaminase 2 Does Not Rescue the Phenotypic Deficits Observed in R6/2 and zQ175 Mouse Models of Huntington's Disease
Source: PLoS One. 2014 Jun 23;9(6):e99520. doi: 10.1371/journal.pone.0099520 (PMC4067284; doi:10.1371/journal.pone.0099520)

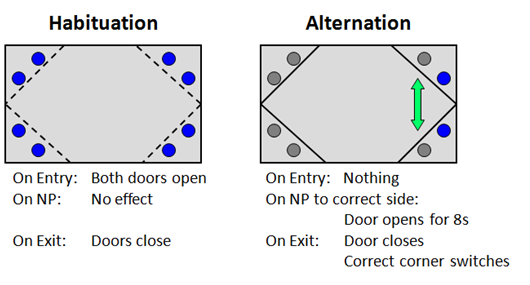

Supplement: Figure S1 — Graphical depiction of cage plan and water access protocol during Habituation and Alternation phases. Shaded circles signal the armed receptacles (NP: Nosepoke). (TIF) [file pone.0099520.s001.tif]
