# Supplementary material for: Genetic Deletion of Transglutaminase 2 Does Not Rescue the Phenotypic Deficits Observed in R6/2 and zQ175 Mouse Models of Huntington's Disease
Source: PLoS One. 2014 Jun 23;9(6):e99520. doi: 10.1371/journal.pone.0099520 (PMC4067284; doi:10.1371/journal.pone.0099520)
Supplement: Table S1 — Effect of TG2 deletion on HD mouse models: comparison of present results to previously published ones. (DOCX) [file pone.0099520.s002.docx]

|  | **Present study - Menalled et al., 2014** | | **Mastroberardino et al., 2002^16^** | **Bailey and Johnson, 2004^17^** | **Bailey and Johnson, 2006^33^** |
| --- | --- | --- | --- | --- | --- |
| **HD mouse model** | R6/2 TG2 -/-  vs.  R6/2 TG2 +/+ | zQ175 Tg2 -/-  vs.  zQ175 TG2 +/+ | R6/1 TG2 -/-  vs.  R6/1 TG2 +/+ | R6/2 TG2 -/-  vs.  R6/2 TG2 +/+ | R6/2 TG2 -/-  vs.  R6/2 TG2 +/+ |
| **CAG size** | ~247 | ~187 | n.r. | 155-175 | 155-175 |
| **Outcome measures evaluated** | |  |  |  |  |
| **Body weight** | ↓(transient) | n.s. | n.r. | n.s. | n.s. |
| **Survival** | N.S. | N.S. up to 12 mo | ↑ | ↑ | ↑ |
| **Locomotor activity** | N.S. | N.S. | n.r. | N.S. | N.S. |
| **Rotarod performance** | n.m. | n.m. | ↑ | ↑ | ↑ |
| **Cognition** | N.S. | n.s | n.r. | n.r. | n.r. |
| **Gene expression dysregulation** | N.S. | N.S. | n.r. | n.r. | n.r. |
| **Brain volume** | N.S. | N.S. | n.r. | N.S. | n.r. |
| **Aggregation** | N.S. (Seprion ELISA) | N.S. (Seprion ELISA) | ↑ | ↑ | n.r. |
| **Other observations** | - | - | ↑ calbindin levels, ↓ abnormal neurons (striatal neurons with condensation) | No change in number of neurons | - |

Abbreviations: N.S.: no significant effect; n.m.: not measured; n.r.: not reported; mo: months of age.
